# Characterization of the Promoter Region of Biosynthetic Enzyme Genes Involved in Berberine Biosynthesis in *Coptis japonica*

**DOI:** 10.3389/fpls.2016.01352

**Published:** 2016-09-02

**Authors:** Yasuyuki Yamada, Tadashi Yoshimoto, Sayumi T. Yoshida, Fumihiko Sato

**Affiliations:** Laboratory of Molecular and Cellular Biology of Totipotency, Division of Integrated Life Science, Graduate School of Biostudies, Kyoto UniversityKyoto, Japan

**Keywords:** berberine, *Coptis japonica*, WRKY, bHLH, gene promoter, isoquinoline alkaloids

## Abstract

The presence of alkaloids is rather specific to certain plant species. However, berberine, an isoquinoline alkaloid, is relatively broadly distributed in the plant kingdom. Thus, berberine biosynthesis has been intensively investigated, especially using *Coptis japonica* cell cultures. Almost all biosynthetic enzyme genes have already been characterized at the molecular level. Particularly, two transcription factors (TFs), a plant-specific WRKY-type TF, CjWRKY1, and a basic helix-loop-helix TF, CjbHLH1, were shown to comprehensively regulate berberine biosynthesis in *C. japonica* cells. In this study, we characterized the promoter region of some biosynthetic enzyme genes and associated *cis*-acting elements involved in the transcriptional regulation via two TFs. The promoter regions of three berberine biosynthetic enzyme genes (*CYP80B2*, *4*′*OMT* and *CYP719A1*) were isolated, and their promoter activities were dissected by a transient assay involving the sequentially truncated promoter::*luciferase* (*LUC*) reporter constructs. Furthermore, transactivation activities of CjWRKY1 were determined using the truncated promoter::*LUC* reporter constructs or constructs with mutated *cis*-elements. These results suggest the involvement of a putative W-box in the regulation of biosynthetic enzyme genes. Direct binding of CjWRKY1 to the W-box DNA sequence was also confirmed by an electrophoresis mobility shift assay and by a chromatin immunoprecipitation assay. In addition, CjbHLH1 also activated transcription from truncated *4*′*OMT* and *CYP719A1* promoters independently of CjWRKY1, suggesting the involvement of a putative E-box. Unexpected transcriptional activation of biosynthetic enzyme genes via a non-W-box sequence and by CjWRKY1 as well as the possible involvement of a GCC-box in berberine biosynthesis in *C. japonica* are discussed.

## Introduction

Higher plants produce a large variety of secondary metabolites, which are commonly classified as phenylpropanoids, aromatic polyketides, terpenoids, and alkaloids. These structurally diverse chemicals help protect the plant against pathogens or herbivore attacks and attract pollinators and are also utilized by humans as dyes, flavorings, and pharmaceuticals (Pichersky and Gershenzon, 2002). In particular, alkaloids, which are nitrogen-containing compounds, are often used as important pharmaceuticals, stimulants and narcotics due to their strong biological activity (Facchini, 2001). However, the biosynthetic pathways of many alkaloids and the regulatory mechanisms of their biosynthesis are largely uncharacterized because the distribution of alkaloids is limited to specific plant species.

**FIGURE 1:**
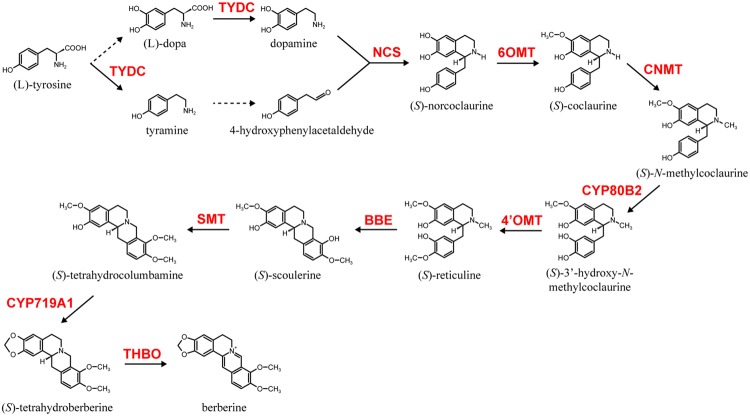
**The berberine biosynthetic pathway in *Coptis japonica*.** Berberine biosynthetic enzymes abbreviations are noted as follows: NCS, (*S*)-norcoclaurine synthase; 6OMT, (*S*)-norcoclaurine 6-*O*-methyltransferase; CNMT, (*S*)-coclaurine-*N*-methyltransferase; CYP80B2, (*S*)-*N*-methylcoclaurine 3′-hydroxylase; 4′OMT, (*S*)-3′-hydroxy-*N*-methylcoclaurine-4′-*O*-methyltransferase; BBE, berberine bridge enzyme; SMT, (*S*)-scoulerine-9-*O*-methyltransferase; CYP719A1, (*S*)-canadine synthase; THBO, (*S*)-tetrahydroprotoberberine oxidase. Broken lines indicate uncharacterized enzyme reactions.

Among alkaloid biosynthesis, the biosynthetic pathways of several alkaloids have been well investigated at the molecular level due to their chemical uniqueness as well as their economic importance (Dewey and Xie, 2013; Hagel and Facchini, 2013; Sato, 2013; Zhu et al., 2014). Examples of these alkaloids include nicotine in *Nicotiana tabacum* (Solananceae), monoterpenoid indole alkaloids (MIAs), vinblastine and vincristine in *Catharanthus roseus* (Apocynaceae), isoquinoline alkaloids (IQAs), berberine in *Coptis japonica* (Ranunculaceae), sanguinarine in *Eschscholzia californica* (Papaveraceae) and morphine in *Papaver somniferum* (Papaveraceae). Among these alkaloids, the biosynthesis of berberine, which is relatively broadly distributed in the plant kingdom and often produced in cultured cells, has been the most intensively investigated (Sato and Yamada, 1984; Ikuta and Itokawa, 1988; Hagel and Facchini, 2013; Sato, 2013). Thus, genes involved in berberine biosynthesis in cultured *C. japonica* cells have been identified and thoroughly characterized (Sato, 2013). These include genes involved in the condensation of dopamine and 4-hydroxyphenylacetaldehyde to (*S*)-norcoclaurine by (*S*)-norcoclaurine synthase (NCS; Minami et al., 2007), the conversion of (*S*)-norcoclaurine to (*S*)-reticuline by the sequential reactions of (*S*)-norcoclaurine 6-*O*-methyltransferase (6OMT; Sato et al., 1994; Morishige et al., 2000), (*S*)-coclaurine-*N*-methyltransferase (CNMT; Choi et al., 2002), (*S*)-*N*-methylcoclaurine 3′-hydroxylase (CYP80B2; Ikezawa et al., 2003), and (*S*)-3′-hydroxy-*N*-methylcoclaurine-4′-*O*-methyltransferase (4′OMT; Morishige et al., 2000), then (*S*)-reticuline to berberine by berberine bridge enzyme (BBE; Minami et al., 2008), (*S*)-scoulerine-9-*O*-methyltransferase (SMT; Takeshita et al., 1995), (*S*)-canadine synthase (CYP719A1; Ikezawa et al., 2003) and (*S*)-tetrahydroprotoberberine oxidase (THBO; Matsushima et al., 2012; **Figure 1**).

Using cultured *C. japonica* cells, specific transcription factors (TFs) involved in berberine biosynthesis have been isolated and characterized because TFs are key for the regulation of gene expression. Specifically, two general transcriptional activators, CjWRKY1 and the non-MYC2-type CjbHLH1, were shown to regulate the expression of almost all of the berberine biosynthetic enzyme genes present in *C. japonica* cells (Kato et al., 2007; Yamada et al., 2011a). Involvement of a WRKY-type TF in IQA biosynthesis has also been confirmed by the heterologous expression of the *Arabidopsis WRKY1* gene in *E. californica* (Papaveraceae) and by the expression of native *PsWRKY* in *P. somniferum* (Apuya et al., 2008; Mishra et al., 2013); these WRKYs are group I type and contain two WRKY domains, in contrast to CjWRKY1, which belongs to the IIc group and contains a single WRKY domain. Involvement of the non-MYC2-type basic helix-loop-helix (bHLH) TF in IQA biosynthesis was also confirmed by the identification of two CjbHLH1 homologs in *E. californica*, EcbHLH1-1 and EcbHLH1-2, which were shown to participate in the regulation of sanguinarine, a benzophenanthridine alkaloid (Yamada et al., 2015).

Characterization of nicotine and MIA biosynthesis pathways allowed the identification of other families of TFs such as AP2/ERF, an MYC2-type bHLH and WRKY that are involved in biosynthesis regulation (Yamada and Sato, 2013; Zhou and Memelink, 2016). Whereas no AP2/ERF-type TFs have been reported to play a role in IQA biosynthesis so far, octadecanoid derivative responsive *Catharanthus* AP2-domain 3 (ORCA3) and NtERF189, which belong to the Group IX AP2/ERF subfamily, were isolated and characterized as transcriptional activators in MIA and nicotine biosynthesis, respectively (van der Fits and Memelink, 2000; van der Fits and Memelink, 2001; Shoji et al., 2010). Nicotine and MIA biosynthesis are also regulated by *Arabidopsis* MYC2-type bHLH TFs, such as NbbHLH1, NtMYC2, and CrMYC2, which show little similarity to CjbHLH1 and function in a jasmonate (JA) signaling cascade together with the CORONATINE INSENSITIVE 1 (COI1) and Jasmonate-ZIM domain proteins (JAZs) (Todd et al., 2010; Shoji and Hashimoto, 2011; Yamada et al., 2011b; Zhang et al., 2011, 2012; De Geyter et al., 2012). In addition to ERF and MYC2-type bHLH, WRKY proteins such as CrWRKY1 (which belongs to a different group than CjWRKY1) were also found to be involved in the regulation of MIA biosynthesis (Suttipanta et al., 2011).

These differences in TF activity found in alkaloid biosynthesis may reflect biosynthetic pathway differences or simply be an artifact resulting from the lack of characterization. Because TFs regulate gene expression through the specific binding to *cis*-acting elements in the promoter region of target genes, we aimed to characterize the promoter sequences of biosynthetic enzyme genes. Previous work indicates that WRKY proteins can specifically recognize the W-box DNA sequence motif (TTGACC/T), while bHLH and AP2/ERF TFs mainly recognize the E-box hexanucleotide consensus sequence (CANNTG) and the GCC-box DNA sequence motif (CGCCGCC), respectively (Atchley and Fitch, 1997; Rushton et al., 2010; Mizoi et al., 2012). We isolated the promoter sequences of three berberine biosynthetic enzyme genes (*CjCYP80B2, Cj4*′*OMT, and CjCYP719A1)* and found that CjWRKY1 activated the *CYP80B2* promoter and CjbHLH1 activated the *4*′*OMT* and *CYP719A1* promoters in a transient luciferase (LUC) reporter assay (Kato et al., 2007; Yamada et al., 2011a); we then characterized these promoter sequences and their interaction with CjWRKY1 and CjbHLH1.

In this study, we predicted several *cis*-acting elements such as W-box, E-box and GCC-box motifs in the *CYP80B2*, *4*′*OMT*, and *CYP719A1* promoter regions and characterized the role of these elements using a *trans*-activation assay involving deletion/mutation *CYP80B2* promoters and co-expression with CjWRKY1. Similarly, the effects of CjbHLH1 expression were investigated using deleted *4*′*OMT* and *CYP719A1* promoters. Our results suggest the involvement of a putative W-box in the regulation of biosynthetic enzyme genes via CjWRKY1 and the involvement of a putative E-box via a CjbHLH1 function. Additionally, we found unexpected transcriptional activation of biosynthetic enzyme genes via non-W-box sequence interaction with CjWRKY1 and the possible involvement of a GCC-box in berberine biosynthesis in *C. japonica*. These results suggest that more research is needed to obtain additional data regarding the transcriptional regulation of alkaloid biosynthesis.

## Materials and Methods

### Plant Material

Suspensions of cultured *C. japonica* cells overexpressing the *CjSMT* gene (156-S cells) were grown in Linsmaier-Skoog (LS; Linsmaier and Skoog, 1965) medium (pH 5.7) containing 3% sucrose, 10 μM 1-naphthylacetic acid (NAA), and 10 nM benzyladenine (BA) on a gyratory shaker (90 rpm) at 23°C in the dark (Sato and Yamada, 1984; Sato et al., 2001).

### Vector Construction

Cauliflower mosaic virus (CaMV) 35S::*GUS*, 35S::*CjWRKY1*, 35S::*CjbHLH1*, *CYP80B2* promoter::*Photinus pyralis (Pp) LUC*, *4*′*OMT* promoter::*PpLUC*, *CYP719A1* promoter::*PpLUC* and 35S::*Renilla reniformis (Rr) LUC* plasmid vectors were constructed as previously described (Kato et al., 2007; Yamada et al., 2011a). Truncated promoter::*LUC* plasmid vectors were constructed in a similar manner as the full length promoter::*LUC* vectors. The *BamH*I/*Sal*I DNA fragments of truncated promoters obtained by PCR were inserted into the upstream region of the *PpLUC* gene. The tandem repeats of the 163 bp *CYP80B2* promoter::*PpLUC* plasmid vector were constructed as follows: the *BamH*I/*Sal*I fragment containing a TATA-box derived from the 35S promoter (5′-GGATCCGCAAGACCCTTCCTCTATATAAGGAAGTTCATTTCATTTGGAGAGGACGTCGAC-3′) was first inserted into the *PpLUC* vector. Next, the 163 bp *CYP80B2* promoter fragments were inserted into the upstream region of the TATA-box using the *Hind*III/*BamH*I and *Xho*I/*BamH*I restriction enzymes. Mutant plasmids were then generated by inverse PCR-based site-directed mutagenesis using a KOD-plus DNA polymerase (Toyobo, Osaka, Japan).

### Dual-LUC Reporter Assay

Promoter activities were assayed using a Dual-Luciferase reporter assay system (Promega, Madison, WI, USA) in *C. japonica* protoplasts isolated from 156-S cells cultured for 2–3 weeks. Transformation was performed with polyethylene glycol (PEG) as previously described (Yamada et al., 2011a) using 4 μg of promoter::*PpLUC* plasmids as reporter constructs, 0.025 μg of 35S::*RrLUC* plasmid as a reference construct and with or without 5 μg of 35S::*GUS*, 35S::*CjWRKY1* or 35S::*CjbHLH1* plasmid as an effector construct. Luciferase activities were measured with a Lumat LB 9507 luminometer (Berthold, Bad Wildbad, Germany) after incubation for 24 h at 24°C in the dark. All experiments were performed with three independent biological replicates.

### Electrophoresis Mobility Shift Assay (EMSA)

To determine the direct DNA-binding activity of CjWRKY1 by EMSA, GST-CjWRKY1 recombinant proteins were expressed in *Escherichia coli* BL21(DE3) from full-length cDNAs of *CjWRKY1* or its mutants cloned into a pGEX-6P-1 vector (GE Healthcare, Piscataway, NJ, USA) at the *BamH*I and *Not*I sites. Protein expression was induced with 0.1 mM isopropyl-β-D-thiogalactoside (IPTG) for 6 h at 28°C. GST-CjWRKY1 proteins were extracted from *Escherichia coli* cells and purified using Glutathione Sepharose 4B (GE Healthcare, Uppsala, Sweden).

The double-stranded DNA probes for EMSA were prepared from biotin-labeled sense and antisense nucleotides annealed in TEN buffer [10 mM Tris-HCl (pH 8.0), 1 mM EDTA, and 0.1 mM NaCl] for 5 min at 95°C and cooled at room temperature. For EMSA, biotin-labeled probes (20 fmol) and the purified recombinant proteins (3 μg) were mixed with 1x binding buffer [1 mM MgCl_2_, 0.5 mM EDTA, 0.05 μg/μl poly(dI-dC)] from a LightShift Chemiluminescent EMSA Kit (Thermo Fisher Scientific, Rockford, IL, USA). After 20 min of incubation at 4°C, the reaction mixtures were separated on a 5% polyacrylamide gel and transblotted onto Hybond-N+ nylon transfer membranes (GE Healthcare, Buckinghamshire, UK). The shifts in biotinylated probes were detected using a Chemiluminescent Nucleic Acid Detection Module Kit (Thermo Fisher Scientific, Rockford, IL, USA) and using the ImageQuant LAS4010 imaging software (GE Healthcare, Buckinghamshire, UK).

### Chromatin Immunoprecipitation (ChIP) Assay

To determine the *in vivo* DNA-binding activity of CjWRKY1 using a ChIP assay, approximately 4 × 10^6^ protoplasts isolated from 2- to 3-week-old 156-S cultured cells were transfected with 300 μg of 35S::*sGFP* (as vector control) or 35S::*CjWRKY1-sGFP* plasmid vector and incubated for 24 h at 24°C in the dark. Then, protoplasts were suspended in 1 ml of W5 solution (154 mM NaCl, 125 mM CaCl_2_⋅2H_2_O, 5 mM KCl, 5 mM glucose, pH 5.8) containing 1% formaldehyde for 10 min to allow cross-linking. Then, immunoprecipitation of chromatin was performed as previously described (Yamada et al., 2011a) with Dynabeads Protein G (Invitrogen, Oslo, Norway) and 5 μl of anti-GFP antibodies (A1122, Invitrogen, Carlsbad, CA, USA). The purified chromatin was subjected to PCR for 33 cycles with specific primer pairs for the detection of genomic regions corresponding to the promoter and the control.

## Results

### Prediction of Putative *Cis*-acting Elements in the Promoter Regions of *CYP80B2*, *4*′*OMT*, and *CYP719A1* Genes

**FIGURE 2:**
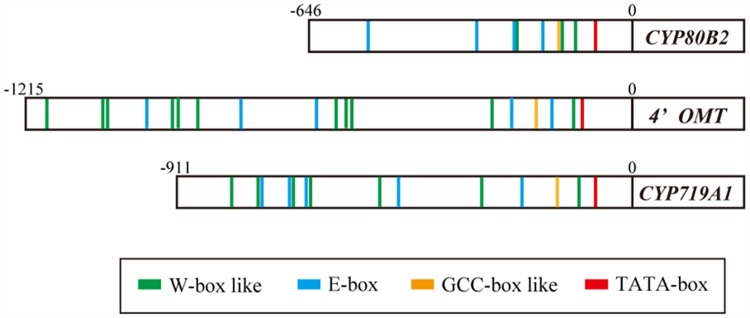
**Putative *cis*-acting elements in the promoter region of *CYP80B2*, *4*′*OMT*, and *CYP719A1* genes.** Putative *cis*-acting elements were predicted by PLACE searches. Red boxes indicate putative TATA-box. Green, blue and yellow boxes indicate predicted W-box, E-box, and GCC-box-like elements, respectively.

The putative *cis*-acting elements in the promoter region of berberine biosynthetic enzyme genes were characterized using 646, 1215, and 911 bp from the 5′ upstream regions of the *CYP80B2*, *4*′*OMT*, and *CYP719A1* genes isolated from *C. japonica* genomic DNA using the TAIL PCR method (**Figure 2**; Kato et al., 2007; Yamada et al., 2011a). The results obtained querying the plant *cis*-acting regulatory DNA elements (PLACE) database (Higo et al., 1998) suggested a putative TATA-box located 76 bp upstream from the translation start site (position -76) in the *CYP80B2* promoter, at the position -107 in the *4*′*OMT* promoter and at the position -78 in the *CYP719A1* promoter. PLACE searches also showed three putative W-box-like elements (TGACT) at positions -227, -138, and -116, one GCC-box-like element (GCAGCC) at position -148, and four E-box elements (CANNTG) at positions -526, -319, -231, and -177 in the *CYP80B2* promoter (Supplementary Figure S1), although CjbHLH1 did not *trans*-activate this promoter sequence (Yamada et al., 2011a).

Plant *cis*-acting regulatory DNA elements queries further showed 11 W-box-like elements (TGACN) at positions -1177, -1052, -1043, -930, -910, -873, -600, -575, -565, -285, and -127, five E-box elements at positions -969, -780, -638, -232, and -155, and one GCC-box-like element (GCCACC) at position -201 in the *4*′*OMT* promoter (Supplementary Figure S2). The *CYP719A1* promoter showed seven W-box-like elements at positions -796, -749, -682, -653, -506, -302, and -107, five E-box elements at -740, -686, -657, -474, and -232, and one GCCACC at -146 (Supplementary Figure S3). These predictions suggested that the expression of *CYP80B2*, *4*′*OMT*, and *CYP719A1* genes may be regulated via several coordinating *cis*-elements and interacting *trans*-acting factors such as CjWRKY1, CjbHLH1, and unknown AP2/ERF TFs.

To investigate whether these putative *cis*-acting elements in the *CYP80B2*, *4*′*OMT*, and *CYP719A1* promoters are crucial for promoter activity, we constructed plasmids containing sequential truncations of each gene, fused them with a reporter gene, and measured their transcriptional activity in *C. japonica* protoplasts with the 35S promoter::*RrLUC* vector as a reference plasmid for transfection control. Specifically, the *CYP80B2* promoter deleted to -418, -217, -127, and -82, the *4*′*OMT* promoter deleted to -581, -342, and -164, and the *CYP719A1* promoter deleted to -708, -479, and -254 were constructed and used for the dual-LUC reporter assay.

**FIGURE 3:**
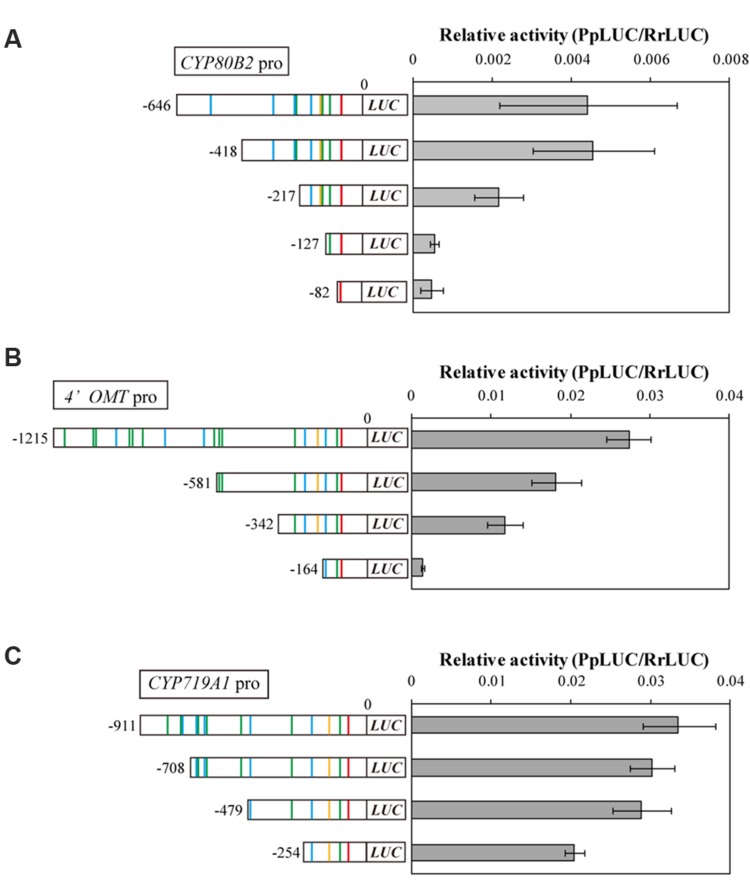
**The relative promoter activity of truncated *CYP80B2***(A)**, *4*′*OMT***(B)**, and *CYP719A1***(C)** constructs.** Reporter plasmid vectors consisted of deleted promoter regions upstream of the *LUC* gene. Vectors were introduced into *C. japonica* protoplasts and relative LUC activities were measured by a dual-LUC reporter assay. The values are the averages of three biological transfections. The data are represented as the mean ± SD.

As shown in **Figure 3A**, the relative LUC activity of the -418 *CYP80B2* promoter was almost equal to the full length (-646) *CYP80B2* promoter, whereas the activities of the -217, -127, and -82 *CYP80B2* promoters were considerably and significantly decreased. The -581 and -342 deletions of the *4*′*OMT* promoter showed a gradual decrease of activity in comparison with the full-length (-1215) promoter; the -164 *4′OMT* promoter showed almost no activity (**Figure 3B**). However, the LUC activity was relatively high for the full length (-911) *CYP719A1* promoter, although the -254 construct showed a slight decrease (**Figure 3C**). These results indicate that certain regions [i.e., sequences between -418 and -217 (one W-box at -227) or between -217 and -127 (one W-box at -138, one GCC-box at -148 and one E-box at -177)] of the *CYP80B2* promoter, and the 178 bp sequence between -342 and -164 (one W-box at -285, one GCC-box at -201, and one E-box at -232) of the *4*′*OMT* promoter could be important for promoter activities.

### CjWRKY1 and *CYP80B2* Promoter *Trans*-Activation Analysis Through Predicted W-box Elements Using Deletion and W-box-Mutated Promoter Constructs

**FIGURE 4:**
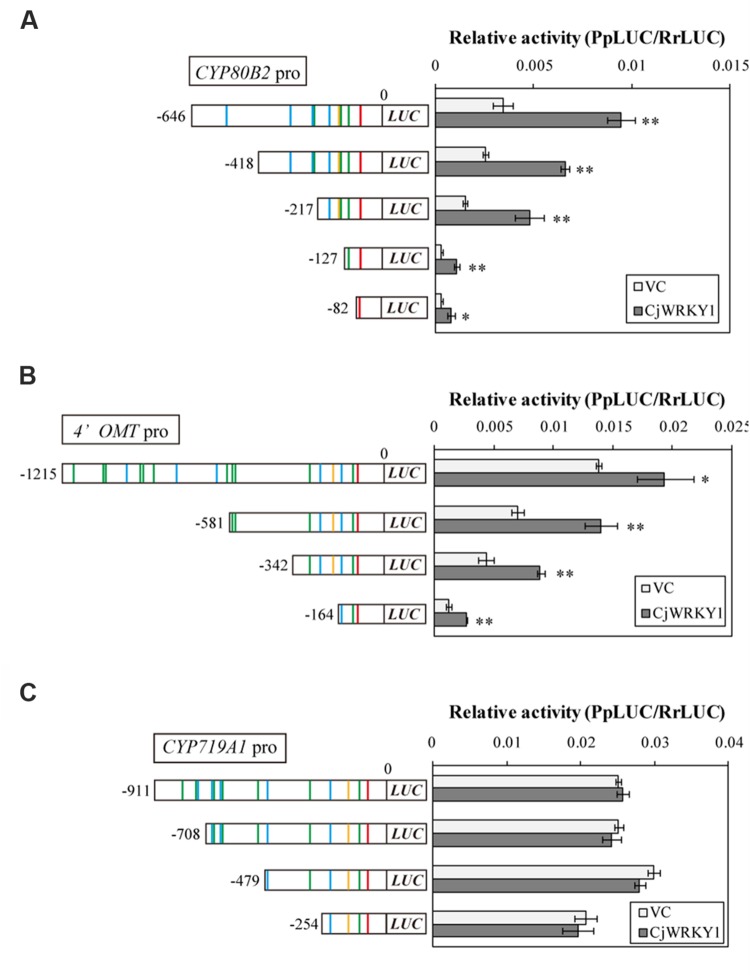
**Transactivation activity of CjWRKY1 in truncated *CYP80B2***(A)**, *4*′*OMT***(B)**, and *CYP719A1***(C)** promoter::*LUC* reporter constructs.** Effector plasmid vectors (35S::*GUS* (vector control; VC) or 35S::*CjWRKY1*) were co-transfected with reporter plasmid vectors containing deleted promoter regions into *C. japonica* protoplasts and relative LUC activities were measured by a dual-LUC reporter assay. The values are the average of three biological transfections, and the data are represented as the mean ± SD, **p < 0.01, *p < 0.05, Student’s *t*-test.

Next, we focused on the transcriptional activity of CjWRKY1 to the truncated promoters. As previously described (Kato et al., 2007), the LUC activity of the full-length *CYP80B2* promoter was significantly enhanced by *CjWRKY1* expression in a transient LUC reporter assay (**Figure 4**). *CjWRKY1* overexpression also significantly activated the LUC activities of the -418, and -217 truncated promoters of the *CYP80B2* gene, while the activities of the -127 and -82 truncated promoters were considerably lowered even with the expression of *CjWRKY1*, suggesting that the W-box element at -138 might be responsible for the CjWRKY1-mediated induction (**Figure 4A**).

The overexpression of *CjWRKY1* also enhanced the LUC activities of the -1215, -581, -342, and -164 truncated promoters of the *4*′*OMT* gene (**Figure 4B**), although the enhancement on the -164 promoter was marginal, which highlights the importance of the W-box located at -285. These results suggest that *trans*-activation of CjWRKY1 on the *CYP80B2* and *4*′*OMT* promoters needs at least one critical putative W-box element (i.e., -138 for *CYP80B2* and -285 for *4*′*OMT*) while others might be not essential. In contrast, the lack of *trans*-activation of the *CYP719A1* gene by CjWRKY1 suggests that those putative W-boxes found in *CYP719A1* might not be functional (**Figure 4C**). Above observation was confirmed by repeated experiments, whereas absolute activity fluctuated at each experiment.

**FIGURE 5:**
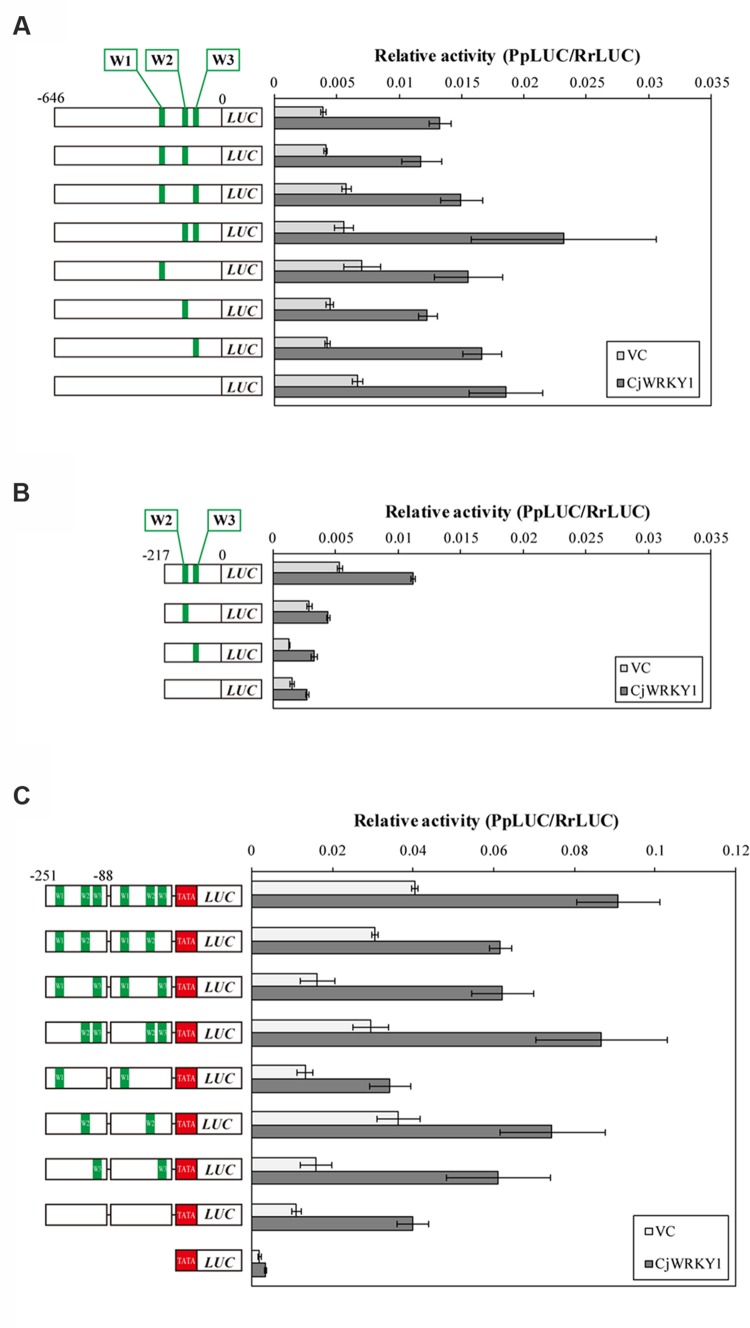
**Mutation of W-box elements in the *CYP80B2* promoter affected the transcriptional activity of CjWRKY1.** Reporter constructs that contain the full length *CYP80B2* promoter **(A)**, the deleted 217 bp *CYP80B2* promoter **(B)** and tandem repeats of the 163 bp *CYP80B2* promoter **(C)** with or without W1, W2, and W3 elements were co-transformed into *C. japonica* protoplasts with effector plasmids. The relative LUC activities were measured by a dual-LUC reporter assay. The values are the average of three biological transfections and the data are represented as the mean ± SD.

Based on the above observation, the three predicted W-box elements found at -227, -138, and -116 in *CYP80B2* gene were named W1, W2, and W3, and their roles in the transcriptional activity of CjWRKY1 were examined using W-box-mutations (TGACT→TTTTT). As shown in **Figure 5A**, single, double and triple mutations of putative W-box elements in the full-length *CYP80B2* gene generated some variability of relative LUC activities, although these effects were marginal. To study the effects of the putative W-box elements, the -217 sequence was used to evaluate the function of the W2 and W3 elements. Mutations in W2, W3, or both and in the -217 *CYP80B2* promoter::*LUC* vector clearly decreased relative LUC activity with or without *CjWRKY1* expression compared to the non-mutated -217 promoter (**Figure 5B**). In particular, the strong reduction of LUC activity in the W2 mutant confirms the important role of the W2 element via CjWRKY1 as previously indicated by the truncated promoter analysis (**Figure 4A**). However, some residual enhancement of LUC activity was still found in the null W-box mutant.

To evaluate the function of the three W-box elements (W1–W3) in the *CYP80B2* promoter in more detail, 163 bp of the *CYP80B2* promoter between -251 and -88 (which includes the W1, W2, and W3 elements) was added to the TATA-box of the CaMV 35S promoter and the *PpLUC* gene, and their activities were measured with a dual-LUC reporter assay. Mutations in W2 and W3, and especially in W2, reduced basal promoter activities without *CjWRKY1* expression compared to the non-mutated promoter. Interestingly, whereas the effect of *CjWRKY1* expression was also reduced with mutations in W2 and W3, considerable LUC activities were still induced by CjWRKY1 (**Figure 5C**). These results confirmed the existence of unexpected LUC activities in the null W-box construct (**Figure 5A**) induced by CjWRKY1 and suggested the possibility that unidentified *cis*-acting elements are directly or indirectly involved in CjWRKY1-mediated induction.

### Detection of Direct Binding of CjWRKY1 to the W-box Element in the *CYP80B2* Promoter

**FIGURE 6:**
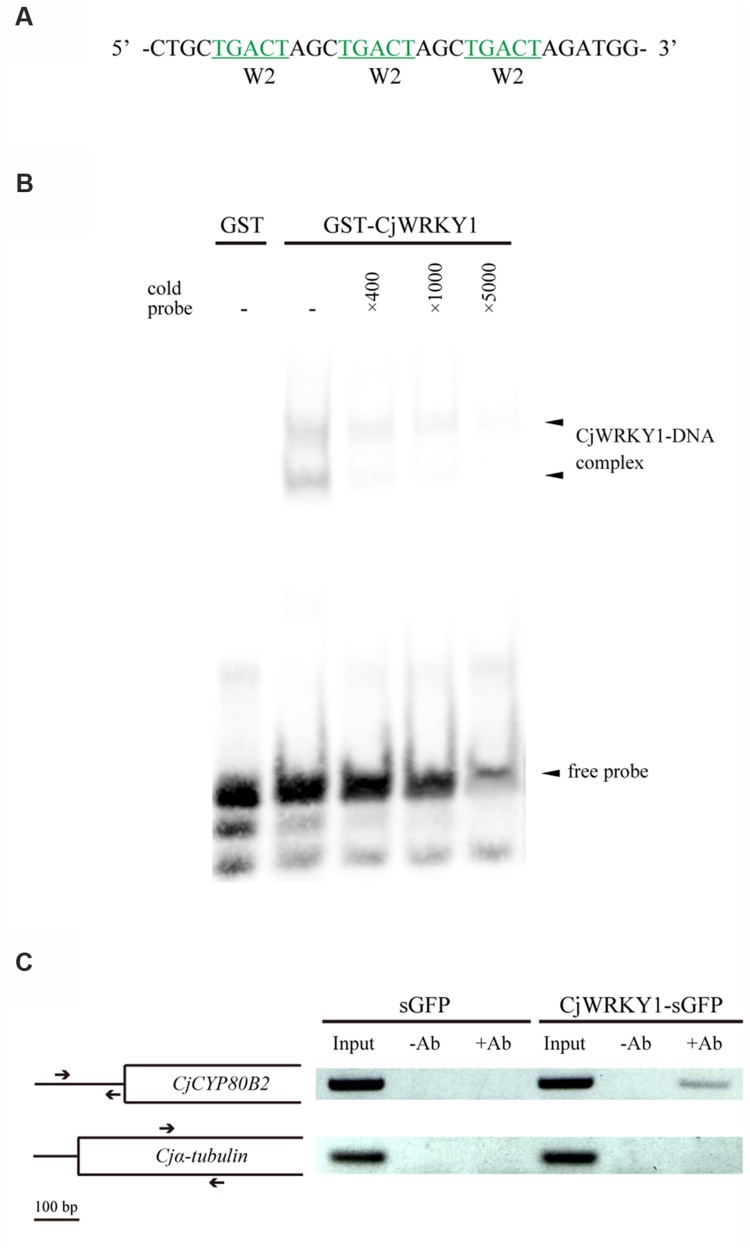
**The binding activity of CjWRKY1 to the *CYP80B2* promoter. (A)** The oligonucleotide sequence used for the EMSA contains three repeats of W-box sequences (TGACT). **(B)** The *in vitro* binding activity of CjWRKY1 protein to the W-box DNA sequence motif was confirmed by EMSA analysis. EMSA was carried out with a purified GST-CjWRKY1 recombinant protein and a biotin-labeled probe. Arrows indicate the shifted bands corresponding to the protein-DNA complexes. **(C)** CjWRKY1 directly binds to the *CYP80B2* promoter region *in vivo*. A ChIP assay was performed with anti-GFP antibodies. The left panel indicates the structure of the coding region of the *CYP80B2* and the α*-tubulin* genes. Arrows indicate specific primer pairs used for PCR. The right panel shows PCR products from immunoprecipitated chromatin and input controls incubated without (-Ab) or with (+Ab) anti-GFP antibodies before immunoprecipitation.

To confirm the direct binding of CjWRKY1 to the predicted W-box elements in the *CYP80B2* promoter, a recombinant GST-fused CjWRKY1 protein was incubated with an oligonucleotide probe that contains three tandem copies of the putative W-box (TGACT) found in the W2 nucleotide sequence (**Figure 6A**). Specific binding was detected by EMSA (**Figure 6B**). Further ChIP analysis using overexpressed CjWRKY1-sGFP proteins and anti-GFP antibodies in *C. japonica* protoplasts (**Figure 6C**) confirmed the amplification of the *CYP80B2* gene promoter region by a specific primer designed to detect the W-box element.

### Detection of CjbHLH1 Transcriptional Activity Using Deleted *4*′*OMT* and *CYP719A1* Promoter::*LUC* Reporter Genes

**FIGURE 7:**
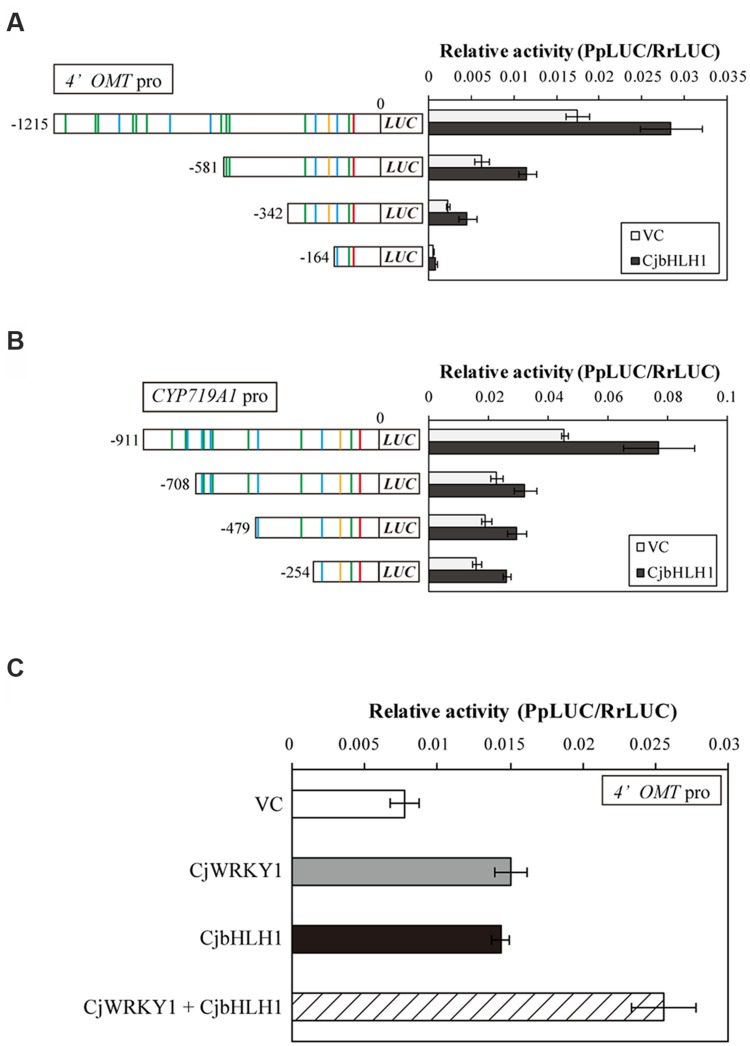
**Transactivation activity of CjbHLH1 in the truncated *4*′*OMT* and *CYP719A1* promoter::*LUC* constructs in comparison with CjWRKY1.** Transactivation activity of CjbHLH1 was measured with truncated *4*′*OMT* **(A)** and *CYP719A1* **(B)** promoter::*LUC* constructs to evaluate the involvement of putative E-box elements. **(C)** The additive effects of CjWRKY1 and CjbHLH1 on the transcription of the *4*′*OMT* gene. 35S::*CjWRKY1* and 35S::*CjbHLH1* effectors were co-transformed into *C. japonica* protoplasts with the full-length *4*′*OMT* promoter::*LUC* construct. The relative LUC activities were measured by a dual-LUC reporter assay. The values are the average of three biological transfections, and the data are represented as the mean ± SD.

In addition to CjWRKY1, CjbHLH1 is also a critical regulator of the IQA biosynthesis pathway in *C. japonica* (Yamada et al., 2011a). Thus, we compared the effects of *CjbHLH1* and *CjWRKY1* overexpression using truncated *4*′*OMT* and *CYP719A1* promoters because the *CYP80B2* promoter was not responsive to *CjbHLH1* overexpression. Both *CjbHLH1* and *CjWRKY1* overexpression similarly enhanced the expression of truncated promoters, although the positive effect of CjWRKY1 on the -164 promoter of the *4*′*OMT* gene was not evident for CjbHLH1, suggesting that the sequence located between -242 and -164 upstream of the *4*′*OMT* gene, which contains one putative E-box element at -232, was critical for CjbHLH1 function. However, truncated *CYP719A1* promoters showed constant induction of LUC activity by CjbHLH1, suggesting that the putative E-box element found in the -254 sequence was still responsive to CjbHLH1 (**Figures 7A,B**).

The interaction of CjWRKY1 and CjbHLH1 was further examined using the *4*′*OMT* promoter because *CYP80B2* was not responsive to CjbHLH1 and *CYP719A1* was not responsive to CjWRKY1 (**Figure 7A**, Yamada et al., 2011a). When the effects of *CjWRKY1* and *CjbHLH1* overexpression were examined with the *4*′*OMT* promoter::*LUC* construct, co-expression of *CjWRKY1* and *CjbHLH1* showed additive enhancement of LUC activity (**Figure 7C**), suggesting that CjWRKY1 and CjbHLH1 function independently through the use of different *cis*-elements.

## Discussion

Secondary metabolites often mediate defense mechanisms against pathogen or herbivore attacks in plants (Pichersky and Gershenzon, 2002); these functions are likely regulated by defense response genes. In fact, TFs found to be associated with alkaloid biosynthesis belong to the WRKY, ERF, and bHLH groups, which are known to be involved in defense responses. In berberine biosynthesis, two TFs, CjWRKY1 and CjbHLH1, were isolated and characterized in *C. japonica* (Kato et al., 2007; Yamada et al., 2011a). Although the regulation of berberine biosynthetic enzyme genes by CjWRKY1 and CjbHLH1 had been previously studied, little is known regarding gene regulation because we lack information on the promoter of these biosynthetic enzyme genes. Thus, in this report, we characterized the promoter regions of three biosynthetic enzyme genes using truncations or mutations of promoter sequences.

Although the lengths of isolated promoter sequences were rather short, the promoter regions of *CYP80B2* (646 bp upstream sequence from the translation initiation site), *4*′*OMT* (1215 bp upstream sequence) and *CYP719A1* (911 bp upstream sequence) managed to activate the *LUC* reporter gene in *C. japonica* protoplasts, and two of them showed an induction of LUC activity by either *CjWRKY1* or *CjbHLH1* co-expression (**Figures 4** and **7**). These results suggest that either isolated promoter regions might not be sufficient or that endogenous expression of TFs may be sufficient for the induction. A clear reduction of transcriptional activity of the 163 bp *CYP80B2* promoter region between -251 and -88 with a mutated E-box (CACGTG→ATCGAC) and GCC-box-like (GCAGCC→GCTTCA) elements suggests that the latter interpretation may be more likely (Supplementary Figure S4). This observation and previous reports suggest that the biosynthetic enzyme genes in the berberine biosynthesis pathway may be regulated by relatively short promoter regions.

Further PLACE and truncation analysis of promoter sequences showed the existence of many putative *cis*-acting elements targeted by WRKY, bHLH and AP2/ERF TFs and that putative elements such as the W-box at the -227 or -138, the GCC-box at -148 and the E-box at -177 of the *CYP80B2* promoter, the W-box at -285, the GCC-box at -201, and the E-box at -232 of the *4*′*OMT* promoter may be important for promoter activities (**Figure 3**). Interestingly, our analysis suggests the involvement of GCC-box and ERF in the regulation of berberine biosynthesis. Although our preliminary transient RNAi assay using available AP2/ERF EST clones (Yamada et al., 2011a) did not show an effect on the transcription of berberine biosynthetic enzyme genes, more careful investigation of this group of TFs is needed because several AP2/ERF TFs such as ORCA3, ERF189, and tomato ERF have been reported to participate in the regulation of MIA biosynthesis in *Catharanthus roseu*s, nicotine biosynthesis in *N. tabacum* and steroidal glycoalkaloid biosynthesis in tomato and potato through binding to GCC-box elements (van der Fits and Memelink, 2000; Shoji et al., 2010; Cárdenas et al., 2016).

The importance of the predicted W-box and E-box elements found in the promoter regions was further confirmed by the LUC activity analysis with truncated promoters and co-expression with *CjWRKY1* and *CjbHLH1*, respectively (**Figures 4** and **7**). More detailed mutation analysis of W-box elements found in the -217 *CYP80B2* promoter sequence further supports the involvement of -138, and -116 W-box elements, although some remaining induction by co-expression with *CjWRKY1* was observed even in the null putative W-box reporter construct.

This remaining activity may be the result of induction by non-conserved W-box sequences or by indirect effects on transcription/translation, including the effects of the reference gene. Because CjWRKY1 was post-transcriptionally regulated by phosphorylation and protein degradation (Yamada and Sato, 2016), overexpression of *CjWRKY1* might interfere/modify the assay system. Furthermore, rice OsWRKY13 is reported to bind not only W-box elements but also the PRE4 element (TGCGCTT), barley SUSIBA2 (HvWRKY46) can bind to a sugar-responsive element (TAAAGATTACTAATAGGAA) as well as the W-box, and NtWRKY12 can bind to the sugar-responsive-like element but not the W-box (Grierson et al., 1994; Sun et al., 2003; Cai et al., 2008; van Verk et al., 2008). While these non-W-box-type elements are not found in the *CYP80B2* promoter, the possibility of finding non-W-box elements via CjWRKY1 needs more careful molecular and biochemical characterization. A potential indirect effect on promoter activity has also been reported in nicotine and MIA biosynthesis (Zhou and Memelink, 2016).

Our study also indicates that promoter activity can be modified by a combination of *cis*-elements. The truncation of the promoter sequences (**Figures 3**, **4** and **7**), the duplicated promoter construct (**Figure 5C**), and the mutation of putative *cis*-elements (**Figure 5B**; Supplementary Figure S4) showed that the effects of putative *cis*-elements are likely to be additive. In fact, when *CjWRKY1* and *CjbHLH1* were co-expressed with the *4*′*OMT* promoter, the effects of these two TFs were additive (**Figure 7C**). This suggests that further careful investigation of promoter sequences and additional information regarding the preferential interaction of *cis*-elements with TFs may allow us to further dissect the regulation of alkaloid biosynthesis gene expression. Zhang et al. (2011) reported that CrMYC2, a MYC2-type bHLH TF involved in the regulation of MIA biosynthesis, regulates the expression of *ORCA3*, an AP2/ERF transcriptional activator of several MIA biosynthesis enzyme genes. Because the regulation of gene expression is complex and varies among alkaloid species, it is clear that we need additional genome information to study it further. In fact, until now, we did not know how the gene expression of *CjWRKY1* and *CjbHLH1* was regulated. Recent progress in next generation sequencing technology can help us obtain whole genome sequences of non-model plants such as *C. japonica* and thus help us elucidate the mechanisms of signal transduction as a result of interactions between TFs and gene promoters in IQA biosynthesis.

## Author Contributions

YY, TY, and SY prepared materials and carried out the preliminary experiments. YY performed the experiments. YY and FS designed the research and wrote the manuscript.

## Conflict of Interest Statement

The authors declare that the research was conducted in the absence of any commercial or financial relationships that could be construed as a potential conflict of interest.
