# Supplementary material for: Characterization of the Promoter Region of Biosynthetic Enzyme Genes Involved in Berberine Biosynthesis in Coptis japonica
Source: Front Plant Sci. 2016 Sep 2;7:1352. doi: 10.3389/fpls.2016.01352 (PMC5009119; doi:10.3389/fpls.2016.01352)
Supplement: Supplementary file 1 [file Presentation_1.PDF]

Supplementary Material

Characterization of promoter region of biosynthetic enzyme genes involved in the berberine biosynthesis in *Coptis japonica*

Yasuyuki Yamada , Tadashi Yoshimoto, Sayumi Yoshida, Fumihiko Sato*

*Correspondence: Fumihiko Sato: fsato@lif.kyoto-u.ac.jp

-646 TCACGGTTAGGAGGACCAAGACCTAATAAACGATCATTGTTGGACTAAAGCCTAATTACC

-586 CTATTATTGGGAGGACCAAAACCTAAATAACCTTATTGGTAAGTTTGAATAGGGTCTTTA

-526 **CATATG**GGTTTATTGATTTTGATCTATGGATGCATTCCAATGTACACATAAGTATTGGTG

-466 AAATCGGTAGAGGAAAGAGAGTAGGAAGAAAAGGAAAAGAGTACGTGAGGGAAGGATGAG

-406 AGAGAAAGGAAAAAGGGAGTTATTCTTTTGCCCTTTTTTCTCTAACTTTTCAAGAAAACC

-346 GGTGATAAGGGTATCCAGTTTCACAGTAATGATGTGG**CAATTG**CTCATTGGTCTGTTACT

-286 TAGTTAGCAGCCAGTAGCTGTCTCCGTAGCATTTTCGATATACGTATTTGCCGTA**CAAAT**

-226 **GACT**GAATTCACAGTTCCCACGATGGTTGTTGTCCTTTCGCACGGGATG**CACGTG**AACTT
W1

-166 GGAATTAACTTAATTCCA**GCAGCC**ATCT**AGTCA**GCAGTTCATTATTCATT**AGTCA**TTTTC
W2 **W3**

-106 GCCCACAGCAAATTTTCTGCCAACAAGCCATAAATAAACAATCATCCGAGTAACCAAAGT

-46 GCTTCACATAGTTGCAAGAACGGAACATAGCTGGATAGGAAAAACT

W-box like E-box GCC-box like

Supplementary Figure S1. Nucleotide sequence of the *CYP80B2* promoter.

-1215 GGTTGGCTCAAAATACGAGGTAAAAGTTGTAAAGGTAC**TGACA**ACAAAAATCACTGAAGA

-1155 GTTGCTGATAGTTTAGTAGTTAGTTGCTGCTTCATTATAGCAAACACATACTACAACATG

-1095 TTTTGATAAACTTGTTTTTTTCATCAAATCCAACGGTTGTCCA**TGACT**GCGA**AGTCA**AAC

-1035 TCGTCAGGTACAGAACGACAGAGTTATGCGCAGTAGTTTACAGGGGAGAGAGAGTGTTTG

-975 CAAGTC**CACGTG**GGCATTGTCTTAAAACAAGGAGAAGGGCAAAAG**TGTCA**AAATCCACGT

-915 TGAAA**AGTCA**AAATTGGAAGTGGGTTACTACATGGGAACAGAT**TGACA**TTATGGGTTACT

-855 AATTGGGAAATTACCCATAAGAATTTCGGCTAAACCAATGCATCAGCTCCACTATAAAAG

-795 TATTTCAATCAACAT**CAAATG**AATGAGAAAAGGGTCGGTCGTGAGCAGTTCTGGACCACA

-735 CAATTTTTTTAAAAAAAAACAAAAAAACCGAAATGTGTGGTCCAAAGCCGCTTTGGACGC

-675 ACTTCGGTCCTAAGCGCTGAGGACCGAAGCCTTTTCC**CAAATG**AATAAGTTATATCCAAA

-615 CATCCTACAACCCCA**TGTCA**ATTAAACATTTTGGCCGAAA**TGTCA**CAGCA**TGTCA**TGTTT

-555 TATTGAAAGCCATATTGTACATATACCCCATAGTAACAAAGTTTGGATTAGGTATTAATT

-495 TGCCAAGGCCCCAAATTGGATAATTCGGAATTCGGAAGTAAATTTGGAGAATTTTGTTTA

-435 AGGAAAAAAAAAATGAGTTGTAAAAAGTGTATATAGATCTCACAAAATATGTTAAAAACAT

-375 GTTAAATGTGGGTAAGTTAATTCGCAAGTAAATTCGGCACACCCCTAATTCACATAATTT

-315 CTAAGACTTTTTTAAAAAAATTGAAGAATTT**TGTCA**CTTTGTGCCAGCCATCGTTTGATAA

-255 ATATTTTCGTCTCATCATTCCAT**CAATTG**CTCTAGCCCATGCACTCCGTGGGCA**GCCACC**

-195 AAAGAAAACGTGCCCCTCGCTTCCGTAGTCCATAGTCCCA**CACTTG**CCTCACTCCATAGT

-135 CCCACACT**TGACA**GCATACTTTCTACCTTATAAGTACATCTCTCACCTATGCATCTATAT

-75 CCAGAGTTGATACCAAGTTTTACACTTGAAAGAAACTAGAAAAGAAATAACGCAAATATT

-15 ACTAAGAGAATTAAG

W-box like E-box GCC-box like

Supplementary Figure S2. Nucleotide sequence of the *4'OMT* promoter.

-911 GTGTTCCCATATATCCGGAAGAGGACTTGTTCGTGTCTTGTAATGTTGTCTTGCTTCCAT

-851 GATAATGTAAAACTTAGTTTTGCTCTAGTTGGTGCTTGCTACTGAAAAATTATTT**AGTCA**

-791 TCAACAGACAGTATCCTAATAAAATGTTAAGAGTATTATGTC**TGACT**GATT**CATTTG**GAC

-731 AACATATACTGCTTTAATTTGTTTATGGTGCTTCTGCAGTTGTGC**CAGGTGTCA**AGTTTG

-671 TCCCAAGGGAATCT**CATCTGACA**TCCATGTATAACAAACGAAAAATGTGGTGTTCTAGAT

-611 GAAAAGGTAGTTAGTGGCTCGGTGGCTGCATGATGAGTGTGCATGTTCTTATGGCAATAA

-551 AGAAGCGTCCAAAGGCCCAACTATCACTTTTTTTTGTTTTTTCGA**TGACA**TGCTCAACTA

-491 TCACAAACGGTGATGCT**CACCTG**TTGGCGAAATGGGCTTTCCATAAGCAACAGAAGACTC

-431 ATCTACCTAGGTGTCTTCTTGATAACTCTGTGGATCCTATGTTTTGCTGATTTTGTTTTA

-371 ATATATATTAAGGTATTATCGAAAAAAAAAACACTAATACTCCTTTGTTTAATTTGGAGA

-311 TGATTTTGT**TGACC**TAGTTGGCTTTCTTATCCATATAATAAGTTTTATCCAATAAACAAA

-251 GAGGATCACGCTTCATCCC**CACGTG**CTTTCCCACGCTACTCACGTTGTCTCACCATATAT

-191 ACCGATGAAATCATGAAAATATGAAATCATTATTCATAGCCATTA**GCCACC**TTCTCATTCCAC

-131 ATAAAGCAGATACTAAATAAT**TGACC**CCCCCACTGTGAACAAAAATCAGATATAAAA

-71 GACTCGAGTGTGCGTTTGGTTTCCTACATAAGTTTCAAAGACGTTGAGAGGTAACAAAAA

-11 CGATAGCAGAA

W-box like E-box GCC-box like

Supplementary Figure S3. Nucleotide sequence of the *CYP719A1* promoter.

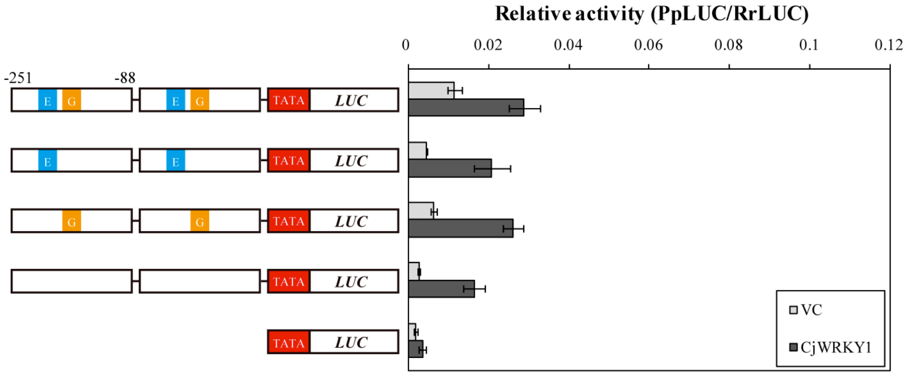

Supplementary Figure S4. Mutation of W-box, G-box and GCC-box elements in the truncated *CYP80B2* promoter affected the transcriptional activity of CjWRKY1.

Reporter constructs which contain tandem repeats of the 163 bp *CYP80B2* promoter with or without G-box and GCC-box elements lacking all W-box elements were co-transformed into *C. japonica* protoplasts with effector constructs. The relative LUC activities were measured by dual-LUC reporter assay. The values are the average of three biological transfections and the data are represented as the mean ± s.d.
